# Evidence of Cnidarians sensitivity to sound after exposure to low frequency noise underwater sources

**DOI:** 10.1038/srep37979

**Published:** 2016-12-21

**Authors:** Marta Solé, Marc Lenoir, José Manuel Fontuño, Mercè Durfort, Mike van der Schaar, Michel André

**Affiliations:** 1 Laboratory of Applied Bioacoustics, Technical University of Catalonia, Barcelona, Spain; 2 INSERM U.1051, Institute of Neurosciences of Montpellier, Montpellier, France; 3 Institute of Marine Sciences, Spanish National Research Council, Barcelona, Spain; 4 Department of Cellular Biology, Faculty of Biology, University of Barcelona, Barcelona, Spain

## Abstract

Jellyfishes represent a group of species that play an important role in oceans, particularly as a food source for different taxa and as a predator of fish larvae and planktonic prey. The massive introduction of artificial sound sources in the oceans has become a concern to science and society. While we are only beginning to understand that non-hearing specialists like cephalopods can be affected by anthropogenic noises and regulation is underway to measure European water noise levels, we still don’t know yet if the impact of sound may be extended to other lower level taxa of the food web. Here we exposed two species of Mediterranean Scyphozoan medusa, *Cotylorhiza tuberculata* and *Rhizostoma pulmo* to a sweep of low frequency sounds. Scanning electron microscopy (SEM) revealed injuries in the statocyst sensory epithelium of both species after exposure to sound, that are consistent with the manifestation of a massive acoustic trauma observed in other species. The presence of acoustic trauma in marine species that are not hearing specialists, like medusa, shows the magnitude of the problem of noise pollution and the complexity of the task to determine threshold values that would help building up regulation to prevent permanent damage of the ecosystems.

From an ecological point of view, the Coelenterates (Cnidarians and Ctenophora) play a central role in marine ecosystems and are key as a food source for different taxa^1^,^2^, as well as a predator of eggs, fish larvae, small planktonic and nektonic prey^3^,^4^. Coelenterates may also help maintaining high biodiversity by preventing the monopolization of biomass by extremely successful competitors^5^. Between Cnidarians, coral reef provide an habitat to a rich diversity of animals, some of them commercially fished species, including crustaceans and other invertebrates, fish, algae and seaweed. Unfortunately, some activities with a huge economic impact as scuba tourism and a large worldwide trade in coral for aquariums and jewellery have resulted in the destruction of many of these resources. In addition, some Scyphozoan medusa are targeted by major fisheries with a strong economic significance^6^,^7^. Cnidaria are used as a model in evolutionary and developmental biology, ecology and morphology. Their structural simplicity, due their basic position on the Metazoan tree, allows revealing basic questions on Cnidaria, which could be treated as a paradigm of general issues, impossible to perform otherwise on more evolved taxa^5^.

Many factors, both from natural or artificial origins, may alter the role of medusa in the marine food web and may have a direct effect on the oceanic environment and economic interests. The effects of influential factors, like the temperature, salinity and food availability on the development of organisms in marine environment have been well described in the literature^8^,^9^,^10^,^11^,^12^,^13^. Recently, levels of introduced anthropogenic underwater sounds have increased significantly over the last century and anthropogenic noise is now recognized as a significant stressor for marine and freshwater fauna. In particular, the European Union Marine Strategy Framework Directive (MSFD) has introduced noise (and other source of energy) in the list of eleven descriptors of the Good Environmental Status of the Ocean^14^. While the noise descriptor does not yet address the effects of noise on marine organisms but provides a standardized protocol to measure and monitor noise levels in EU waters, there is an urgent need to define and quantify the added spatial-temporal variability of acoustic pollution from different sources and identify the resulting short, medium or long-term changes and effects on marine fauna. However, we face the relative lack of information on the sound processing and analysis mechanisms in most marine animals^15^,^16^. While advances have been made in understanding the effects on marine mammals^17^,^18^,^19^,^20^,^21^ and fishes^22^,^23^,^24^, data on the effects on marine invertebrates is still scarce. Much remains to be learned about the hearing or sound-producing capabilities of invertebrates and how they respond to, and are potentially affected by, man-made sounds. However, a few studies on invertebrate sensitivity to noise and possible negative effects after sound exposure have been recently published^25^,^26^,^27^,^28^,^29^,^30^,^31^,^32^,^33^,^34^. A detailed literature review on these effects can be found in recent publications^35^,^36^,^37^.

Ctenophores^38^ and Cnidarians, both in the polyp and the medusa stage, poses sensory organs able to detect vibrations in water associated to prey movement. Hydrozoan and Cubozoan polyps have different types of cells bearing specialized cilia as well, which are located in their tentacles and act as mechanoreceptors^39^,^40^,^41^,^42^. Their function is to inform the animals of the changes in their surrounding environment.

Medusa present receptors that respond to light, touch, gravity, chemicals, sound pressure waves, direction, vibration, and hydrostatic pressure^43^. Sensitivity to sound pressure waves and vibration, which detection is mediated by directionally sensory ciliary hairs, has been inferred from behavioural observations on *Aurelia labiata* under turbulent water. It was shown that the stimulus for this behaviour was vibration or sound pressure waves from the turbulent water above them^44^. Scyphozoan medusae (disc-shaped or “true” jellyfish) are some of the most primitive forms of life to develop pelagic medusa stages with marginal sense organs bearing statocysts^45^. Each Scyphozoan medusa forms numerous small crystals, which are collected in sac-like statocyst located at the distal ends of their rhopalia (Fig. 1). Rhopalia are complex sensory organs, which have been associated with pulsing, swimming and orientation and that act as gravireceptors, which enable medusae to position themselves in an upright posture after tilting^46^. The chemical composition (bassanite calcium sulphate hemihydrate)^47^, the crystallography and the morphology of the crystals (statolith) contained in the statocysts have been examined in some species of Scyphozoan medusa; their differential features were used for taxonomical relationships identification of jellyfish and had led to the development of an age-determination method for jellyfish^48^,^49^,^50^.

Rhopalia are sensitive bodies located around the bell margin in Scyphozoan medusae with a number typically being in multiples of four^51^,^52^. The structure of scyphozoan rhopalia can be seen in Fig. 1. Each rhopalium presents a statocyst at its terminal end (Fig. 1) containing many refractive crystals. The statocyst role is relevant for the gravity-sensing function of the rhopalium; the statocyst together with the adjacent sensory areas work as a gravity organ in Scyphozoan rhopalia^53^,^54^,^55^,^56^,^57^ When the medusa is tilted, gravity pulls up the statocyst, bending the body of the rhopalium, so that cilia on the sensory cells in the sensory plate make contact with or are removed away from the overlying epithelium (hood). The mechanical stimuli trigger the upright positioning behaviour, which occurs by asymmetric contraction of the swimming muscle to restore the balance against the gravitational force^46^.

The aim of the present study was to contribute to a better understanding of the sensitivity to human-generated noise of Scyphozoan medusa by comparatively describing the ultrastructure of two species presents in the Mediterranean Sea (*Cotylorhiza tuberculata (Cepheidae*) and *Rhizostoma pulmo (Rhizostomatidae*) statocyst sensory epithelium after exposure to sound.

## Results

### *Cotylorhiza tuberculata* and *Rhizostoma pulmo* rhopalia morphology

**Figure 1:**
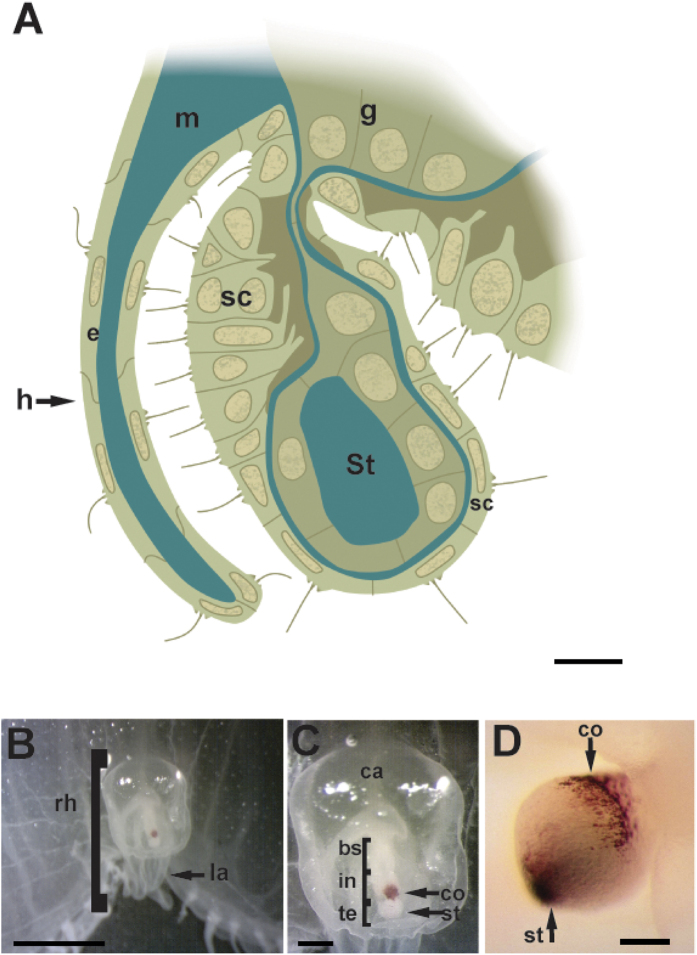
(**A**) Diagram of a longitudinal section through a Sciphozoan medusa rhopalium (**B,C,D**) LM. Aurelia aurita rhopalia photomicrographs. (**A**) The statocyst (at the distal part of the rhopalium) is covered by an epithelium (hood) situated between the bulb (basal part of the rhopalium) and the statocyst. The connection of the bulb and the statocyst is provided with a compact mesoglea. (**B**) Anterior view of the rhopalium in the bell margin. (**C**) Detail from (**B**) There is a mass of sensory cells with a single layer of pigment cells (pigment-cup ocellus) on the oral side near the statocyst. (**D**) Rhopalium showing the statocyst at the terminal end (bs: basal segment, ca: rhopalar canal, co: pigment-cup ocellus, e: epidermis, g: gastrodermis, h: hood, in: intermediate segment, la: lappet, m: mesoglea, rh: rhopalium, sc: statocyst, st: statolith, te: terminal segment). Scale bars: (**A**) = 20 μm. (**B**) = 400 μm. (**C**) = 70 μm. (**D**) = 10 μm.

**Figure 2:**
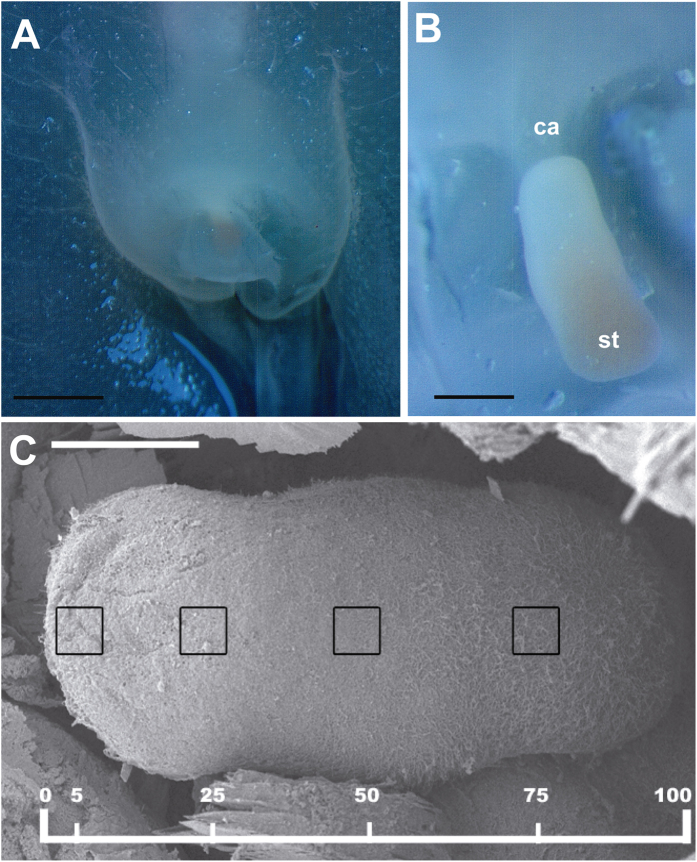
LM (**A,B**) and SEM (**C**). *C. tuberculata* rhopalium structure. (**A**) The rhopalium covered by the hood is visible in its normal position in the bell margin of the medusa. (**B**) The hood has been removed and the photomicrograph shows the complete fixated rhopalium. (**C**) Hair cell bundles count locations on the *C. tuberculata* rhopalium. Hair cells counts were sampled at four predetermined locations: 5, 25, 50 and 75% of the total rhopaliar length. A 900 μm^2^ box was placed at each sampling area and hair cells were counted within each box. (ca: rhopalar canal, st: statocyst) Scale bars: (**A**) = 1 mm. (**B**) = 200 μm. (**C**) = 100 μm.

**Figure 3:**
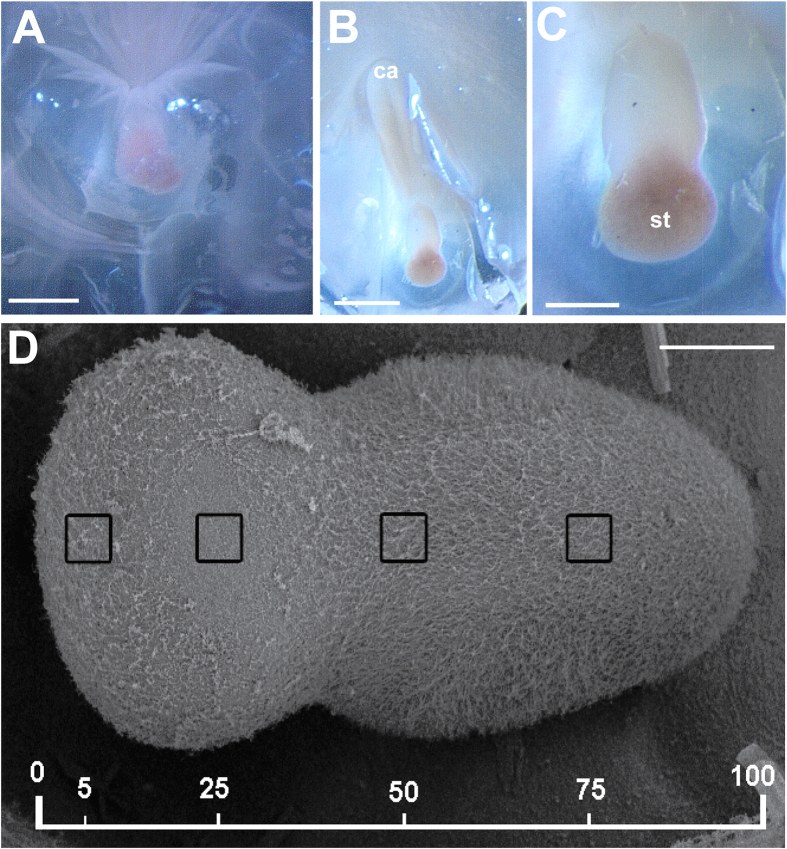
LM (**A,B,C**) and SEM (**D**). R. pulmo rhopalium structure. (**A**) The rhopalium covered by the hood is visible in its normal position in the bell margin of the medusa. (**B**) The hood was removed and the photomicrograph shows the complete fixated rhopalium. (**C**) Detail from (**B**). (**D**) Hair cell bundles count locations on the *R. pulmo* rhopalium. Hair cells counts were sampled at four predetermined locations: 5, 25, 50 and 75% of the total rhopaliar length. A 900 μm^2^ box was placed at each sampling area and hair cells were counted within each box. (ca: rhopalar canal, st: statocyst) Scale bars: (**A**,**B**) = 0, 5 mm. (**C**) = 200 μm. (**D**) = 100 μm.

**Figure 4:**
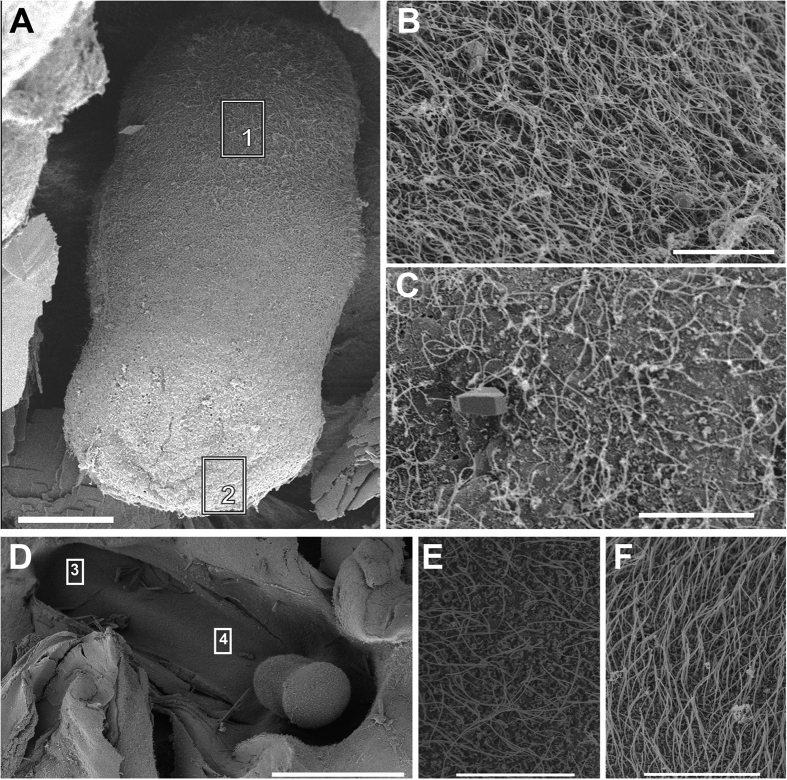
SEM. *C. tuberculata* (**A–C**) and *R. pulmo* (**D–F**) rhopalium. Control animals. (**A**) Rhopalium shows different hair cell densities on its surface. (**B**) Detail from A corresponding square 1. Nearest the hood and on the opposite proximal side of the statocyst the hair cells are more abundant. (**C**) Detail from A corresponding square 2. On the most distal part of the statocyst the sensory cells are more scattered and less abundant. (**D**) Rhopalar canal located next to the rhopalium**. (E,F**) different density of the epithelium that covers the rophalar canal. (**E**) Detail from D corresponding square 3. (**F**) Detail from D corresponding square 4. Scale bars: (**A**) = 100 μm. (**D**) = 500 μm. (**B**,**C**,**E**,**F**) = 20 μm.

**Figure 5:**
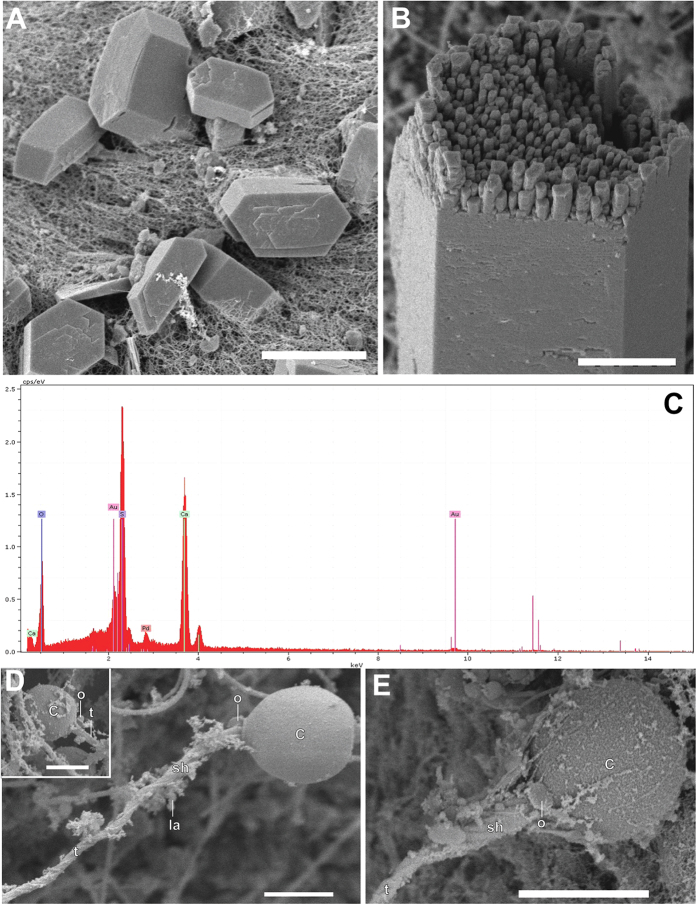
(**A,B,D,E**) SEM. (C) EDX analysis. (A) (C) tuberculata statolith. (B) R. pulmo statolith. The broken crystal allows to see the inner structure of the statolith. (**C**) **Chemical analysis of statolith** shows the mainly presence of calcium, sulphur and oxygen. Au and Pd peaks appears because sample was previously coated with Au-Pd. **(D)*R. pulmo* discharged eurytele cnidocyst**. The operculum is bent up after discharge of the tubule. The shaft bears long. **Insert in (D)** ***R. pulmo* discharged haploneme cnidocyst**. The operculum split off from the capsule during the discharge of the tubule. **(E) *C. tuberculata* discharged eurytele cnidocyst** (c: capsule, o- operculum, sh: shaft, la: lamellae, t: tubule). Scale bars: A, D, E = 5 μm. B = 2 μm.

**Figure 6:**
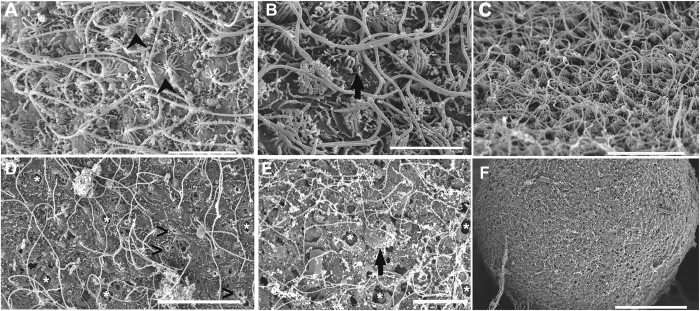
SEM. *C. tuberculata* sensory epithelium. (**A,B,C**) Control animals. (D) animal sacrificed 48 h after sound exposure. (**E,F**) animals sacrificed 96 h after sound exposure. (**A,B,C**) Images of different density areas of sensory statocyst epithelium. (**A,B**) The hair cells bearing nonmotile kinocilia surrounded by a crown of stereocilia are visible (arrows). (**C**) Sensory epithelium on the dense area of the rhopalium. (**D**) fragment of sensory epithelium showing partially or totally extruded hair cells leaving holes (white asterisk) on the epithelium. Some hair cells lost the unique kinocilium (arrowhead) or exhibit bent kinocilia. (**E**) The ejected hair cells (arrow) left holes (asterisk) on the sensory epithelium. The remaining cells presented bent or flaccid kinocilia. (**F**) Large extensions of sensory epithelium presented holes because of the extrusion of the hair cells. Scale bars: (**A**,**C**,**D**) = 5 μm. (**B**) = 1 μm. (**E**) = 10 μm. (**F**) = 20 μm.

**Figure 7:**
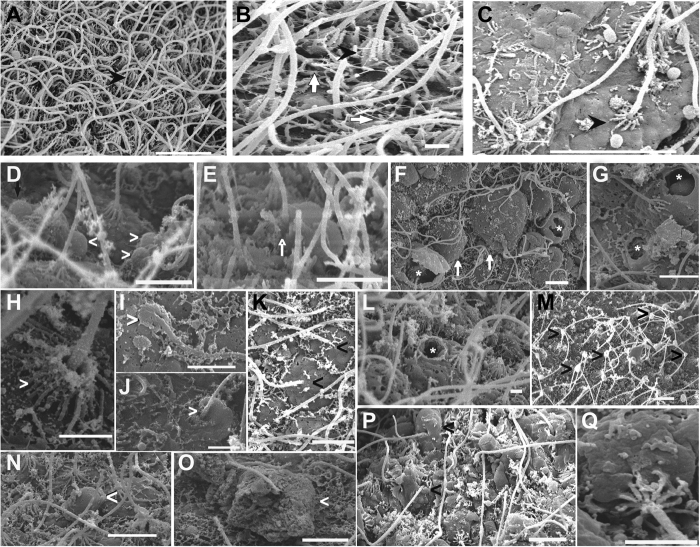
SEM. *C. tuberculata* sensory epithelium. (**A,B,C**) Control animals. (**D,E,H,K,M**) animals sacrificed 48 h after sound exposure. (**F,G,I,J,L,N–Q**) animals sacrificed 96 h after sound exposure. (**A,B,C**) The sensory areas are characterized by nonmotile kinocilia on their surface. Each long straight kinocilium is surrounded by a sort of “folded crater” of stereocilia that seem little fingers around the central shaft (arrowheads). (**A**) Sensory epithelium on the dense area of the rhopalium. (**B**) Detail from (**D**) Arrows show the links between the stereocilia on each hair cell. (**C**) Sensory epithelium on the less dense area of the rhopalium. (**D**) Hair cells presented large bubbles on the apical poles (arrowhead). (**E**) Hair cells presented swollen apical poles (arrow). (**F**) The rupture of the plasma membrane (asterisk) caused by the swelling and the extrusion of the cellular body is shown (arrows). (**G**) Spherical holes on the basis of hair cells are visible (asterisk). (**H**) The crown of stereocilia surrounding the large kinocilium was partially fused (arrowhead). (**I**,**J**) The crown of stereocilia was totally fused (arrowhead). (**K**) The crown of stereocilia surrounding the large kinocilium was lost (arrowhead). (**L**) The loss of the kinocilia and the crown of stereocilia left a hole (asterisk). (**M**) Some hair cells present swollen kinocilia (arrowheads). (**N–Q**) Totally extruded or ejected hair cells remaining in the surface of the epithelium (arrowhead). Scale bars: (**A**), C = 5 μm. (**B,D–G**), (**I**–**P**) = 1 μm. (**H**) Q = 0, 5 μm.

**Figure 8:**
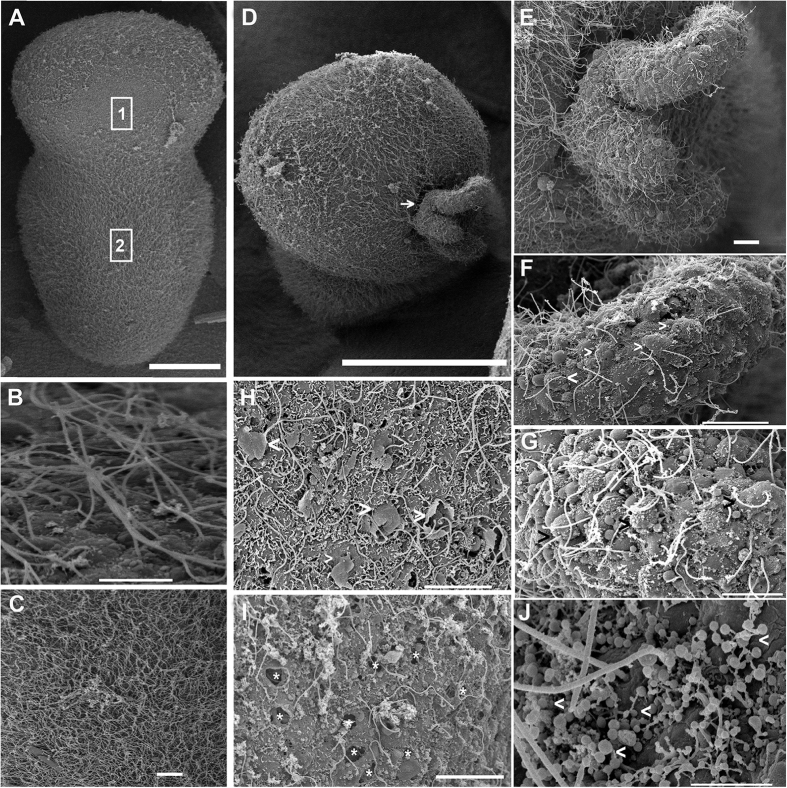
SEM *R. pulmo* sensory epithelium. (**A**–**C**) Control animals. H: animals sacrificed 48 h after sound exposure. (**D–G,I,J**) animals sacrificed 96 h after sound exposure. (**A**) Rhopalium shows different hair cell densities on its surface. (**B**) Detail from A corresponding square 1. On the most distal part of the statocyst the sensory cells are more scattered and less abundant. (**C**) Detail from A corresponding square 2. Nearest the hood and on the opposite proximal side of the statocyst the hair cells are more abundant. (**D**) Images of a rhopalium showing the protrusion of a large fragment of sensory epithelia (arrow). (**E**) Detail from A, shows the fragment of rhopalium sensory epithelium protruded. (**F**) Detail from B. It allows to see that, in the protruded fragment, some hair cells were partially or totally extruded and have lost the crown of stereocilia surrounding the kinocilia (arrowhead). (**G**) Almost the totally of the hair cells from the protruded fragment are extruded on the surface of the sensory epithelium (arrowhead). The hair cells exhibit their unique kinocilium bent and flaccid and the crowns of sterocilia are lost. (**H**) Some hair cells are extruded on the surface of sensory epithelium as a consequence of the rupture of the plasmatic membrane, visible on the base of the hair cells (arrowhead). The kinocilia are bent and flaccid and the crowns of stereocilia are fused or lost. Around the kinocilia, bent and flaccid microvilli are visible. (**I**) At 96 h the totally extrusion of the hair cells left holes in the sensory epithelium surface (asterisk). (**J**) Microvilli show swollen tips (arrowhead). Scale bars: (**A**) = 100 μm. (**D**) = 50 μm. (**C**,**E**–**I**) = 10 μm. (**B**) = 5 μm. (**J**) = 1 μm.

In *C. tuberculata*, between 4 and 8 rhopalia were present in each individual depending on its diameter at the bell margin, hanging downwards between the clefts of the marginal lappets. In all studied *R. pulmo* individuals, 8 rhopalia were present for each individual. The rhopalium of the two species consisted of a proximal bulb and a distal statocyst containing the statolith crystals (Figs 1A, 2 and 3). The statocyst was covered by a layer (hood) situated in the area between the bulb and the statocyst. There are two densely ciliated sensory areas on the epithelium which cover the statocyst, one nearest the hood and other on the opposite proximal side of the statocyst (Figs 1A, 2 and 3). On the most distal part of the statocyst (Fig. 4A–C), coinciding with the area occupied by statolith (Fig. 5A–C), the sensory cells are more scattered and less abundant. Sensory areas are characterized by hair cells carrying nonmotile kinocilia on their surface. Each long straight kinocilium is surrounded by a short crown (“a sort of folded crater”) of stereocilia around the central shaft (Figs 6A–C, 7A–C and 8B-C). The rhopaliar canal, containing the rhopalia, has inner totally covered by a ciliated epithelium (Fig. 4D–F).

As in other rhopaliophoran medusae, the statocysts of these two Sciphozoan medusae are found to be composed of bassanite (calcium sulphate hemihydrate), Fig. 5C. The elemental analysis performed by EDX showed mainly the presence of calcium, sulphur and oxygen, indicating the presence of calcium sulphate. Statocysts mostly consist of an accumulation of single crystals (statoliths) of small size surrounded by gastrodermal cells in both examined species (Fig. 5A,B).

Numerous cnidocysts (organelle used for capture of prey and defense, consisting of a cylindrical capsule, which releases a long tubule upon triggering) covered the marginal lappets and the surface of the *C. tuberculata* and *R. pulmo* rophalium. Two types of cnidocysts, haplonemes and euryteles, were present in the two Sciphozoan medusa species (Fig. 5D,E).

### Ultrastructural analysis of the statocyst sensory epithelium

This study characterized some of the ultrastructural changes that took place following acoustic damage to the Cnidarians statocyst sensory epithelia. Regardless of the species, all exposed individuals presented the same lesions in the statocyst sensory epithelium and the same incremental effects versus time. Damaged hair cells were extruded or missing or presented bent, flaccid or missed kinocilia and stereocilia.

#### Cotylorhiza tuberculata sensory epithelium

After sound exposure (Figs 6D–F and 7D–Q) in comparison with the same tissues from control animals (Figs 6A–C and 7A–C), damage was observed on the sensory epithelium by SEM analysis.

On animals sacrificed 48 h after sound exposure (Figs 6D and 7D,E,H,K,M), the sensory epithelium presented hair cells partially (showing their swollen apical pole) or totally ejected from the sensory epithelium. The apical ciliated apex and part of the cellular body were extruded above the sensory epithelium into the statocyst cavity (Fig. 6D). A considerable number of hair cells had totally lost the unique kinocilium or exhibited bent or swollen kinocilia (Figs 6D and 7M) and the crown of stereocilia surrounding the large kinocilium was lost or totally fused (Fig. 7H,K). The hair cells presented large bubbles (Fig. 7D) onto moderate swollen apical poles (Fig. 7E).

On animals sacrificed 96 hours after sound exposure (Figs 6E,F and 7F,G,I,J,L,N–Q) the lesions reported in the sensory epithelia of the previous groups of exposed animals were present and the effects incremented versus time. Some hair cells presented spherical holes (Fig. 7G) and rupture of the plasma membrane (Fig. 7F) at the base of kinocilia, probably due to the swelling and extrusion of the cellular body. The sensory epithelia presented totally extruded or ejected hair cells (Fig. 7N–Q) leaving large extensions of holes on the sensory epithelia (Fig. 6E,F). The remaining cells exhibited bent or flaccid kinocilia or had lost them (Figs 6E and 7L). The crown of stereocilia of the sensory cells was lost or totally fused (Fig. 7I,J,L).

#### Rhizostoma pulmo sensory epithelium

Serious damage was found in the sensory epithelium from 48 h (Fig. 8H) until 96 h (Fig. 8D–G,I,J) after sound exposure in comparison with the same tissues from control animals (Fig. 8A–C).

Forty eight hours after sound exposure (Fig. 8H), a rupture of the plasma membrane, due the extrusion of the internal cellular material could be seen at the base of the hair cells. As a consequence, ciliated hair cells were partially ejected from the sensory epithelium. Some individuals showed bent and flaccid or lost kinocilia in their hair cells. Sometimes, the crown of stereocilia was found to be fused or totally lost and the epithelium showed remaining holes. The extrusions of hair cells were most evident and the extruded hair cells body was visible on the surface of the sensory epithelium.

On the individuals sacrificed 96 h after sound exposure (Fig. 8D–G,I,J) the same lesions were present, but in addition, the protrusion of large fragments of sensory epithelia left extremely interesting images where the remaining (and almost) extruded hair cells showed bent and flaccid or lost kinocilia (Fig. 8D–G). Large extensions of the epithelium presented holes as a consequence of the total hair cells extrusion (Fig. 8I). Some individuals presented some bent, flaccid microvilli showing swollen tips (Fig. 8J).

### Image and Data analysis

**Figure 9:**
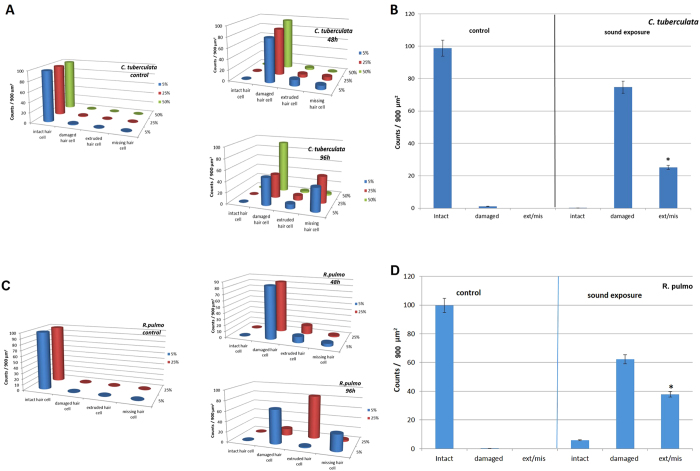
Mean intact hair cell, damaged hair cell, extruded hair cell and missing hair cell at 5, 25 and 50% of the total length of sensory epithelium on rhopalia of *C. tuberculata*. (**A**) and at 5 and 25% of the total length of sensory epithelium on rhopalia of *R. pulmo* (**C**) (48 h and 96 h after sound exposure versus control animals). Note the increase of damaged, extruded and missing cells versus controls with increase of time. **Mean (±SE) intact hair cell, damaged hair cell, extruded/missing hair cells after sound exposure versus control on epithelium rhopalia of *C. tuberculata* (B) (n = 16) and *R. pulmo* (D) (n = 8).** (**B,D**: Each bar is the average over the 3 (**B**) or 2 (**D**) zones with the line indicating the standard error. The percentage was computed by dividing with the total count for each individual sample.)

The abnormal features we identified on the surface of sound-exposure epithelia included damaged hair cell (kinocilium or surrounding sterocilia partially or entirely missing or fused), extruded (hair cell partially extruded of the epithelium) and missing hair cell (hole in the epithelium caused by the total extrusion of the hair cell). The number of damaged, extruded and missing hair cells was computed for each image. In both species, the number of damaged, extruded and missing hair cell increased with time (Fig. 9A,C).

Damage was chosen to be quantified as the percentage of extruded and missing hair cells because these are well-defined and easy-to-compare categories. The presence of extruded cells showed the start of severe damage after sound exposure. All control animals were in good health status (not showing extruded or missing hair cells on the sensory epithelia) and all exposed animals had various degrees of damage; no statistical analysis was therefore performed to test the difference between the two groups. We concluded that statocyst epithelium was affected by exposure to sound in both species (Fig. 9B,D).

**Figure 10:**
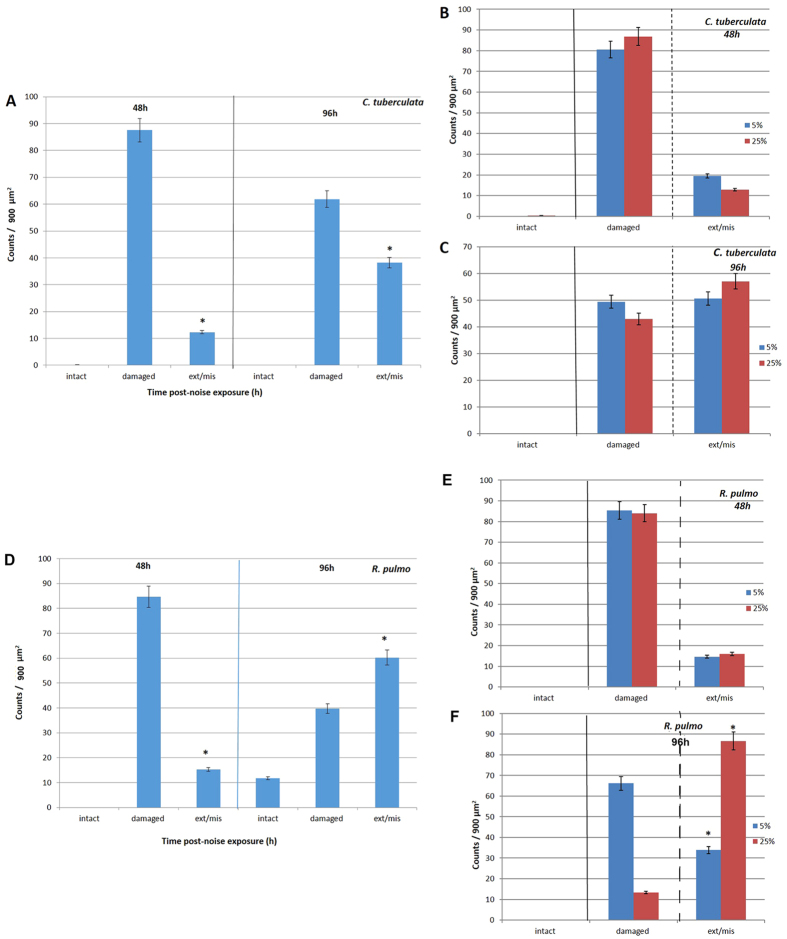
(**A**) Mean (±SE) intact hair cell, damaged hair cell, extruded/missing hair cells at 50% of the total length of sensory epithelium on rhopalia of *C. tuberculata* (48 h versus 96 h after sound exposure) (*p = 0.001; n = 16). (**B**) Differences in percentage of extruded and missing hair cells between 5% and 25% in animals 48 h after sound exposure (*p = 0, 07; n = 16). (**C**) Differences in percentage of extruded and missing hair cells between 5% and 25% in animals 96 h after sound exposure (*p = 0, 14; n = 16). (**D**) Mean (±SE) intact hair cell, damaged hair cell, extruded/missing hair cells at 25% of the total length of sensory epithelium on rhopalia of *R. pulmo* (48 h versus 96 h after sound exposure) (*p = 0.001; n = 8). (**E**) Differences in percentage of extruded and missing hair cells between 5% and 25% in animals 48 h after sound exposure (*p = 0, 66; n = 16). (**F**) Differences in percentage of extruded and missing hair cells between 5% and 25% in animals 96 h after sound exposure (*p = 0, 001; n = 8). Each bar is the average over the 3 (**A–C**) or 2 (**D–F**) zones with the line indicating the standard error. The percentage was computed by dividing with the total count for each individual sample.

After performing statistical tests, the quantification analysis showed that the severity of the lesions, quantified as number of extruded/missing hair cells, increased with time in *C. tuberculata* and *R. pulmo* (p = 0,001) (Fig. 10A,D). The severity of the lesions, quantified as number of extruded/missing hair cells, increased with time.

Regarding the differences between regions, we compared the 5% and 25% regions of the two species (Figs 2C and 3D). The results show that there was no difference in damage between 5% and 25% regions in animals observed 48 h after sound exposure (p = 0,07), nor 96 h after sound exposure in *C. tuberculata* (p = 0,14) (Fig. 10B,C). In the case of *R. pulmo,* there was no difference in damage between 5% and 25% regions in animals observed 48 h after sound exposure (p = 0,66), but there was a difference between 5% and 25% regions in animals observed 96 h after sound exposure (p = 0,001) (Fig. 10E,F).

## Discussion

To the best of our knowledge, this study shows the first published SEM images of the *C. tuberculata* and *R. pulmo* statocyst sensory epithelium. No previous ultrastructural studies have been carried out on these two species. As in other Sciphozoan medusa like *P. periphylla*^48^ or *Nausithoe punctata*^55^, we show that the sensory areas are characterized by nonmotile kinocilia surrounded by a crown of stereocilia that projects in the sea water towards the opposite side of the fold in which these cells lie. As in *P. periphylla*^48^ the density of these ciliated sensory areas differs along the rophalia. There are two densely ciliated sensory areas on the epithelium, which cover the rhopalia, one nearest the hood and other on the opposite proximal side of the statocyst. On the most distal part of the statocyst, the sensory cells are more scattered and less abundant. Since the publication of the first comparative study on the evolution of the statocyst sensory epithelia by Horridge^55^, which described a unique kinocilium surrounded by a crown on stereocilia in different species of Cnidarians, until the description of the most evolved sensory systems among the invertebrates, which present sensory bundles of kinocilia on hair cells in cephalopods^58^,^59^,^60^,^61^, it appears that the role of the kinocilium as a transducer of the sensory signal is fundamental for the mechanoreception process. Some studies analysed the structure and function of the nervous plexus in Sciphozoan^51^,^54^,^62^ and Cubozoan medusa^62^,^63^. Chapman^54^ hypothesized the neurite origin on the intraepithelial flagella in *A. aurita*. The structure, function and evolution of the statocysts and the role of three types of sensory receptors were described by *Aglanta digitale (Hydromedusae*)^64^,^65^. Further analysis at different live stages, including the period of development and aging, are needed for a better description of these sensory ultrastructures in *C. tuberculata* and *R. pulmo*.

Some authors previously reported that statolith of ropaliophoran medusa are composed of basanite (calcium sulphate hemihydrate)^47^,^57^,^66^,^67^. We confirmed that the chemical composition on the *C. tuberculata* and *R. pulmo* statocyst is basanite. In these two species, the statolith mostly consists of an aggregation of single crystals. This statolith structure is common among Scyphozoan medusa^1^,^47^. The statolith crystals are surrounded by gastrodermal cells in Scyphozoan medusa. As in *P. periphylla*^48^ the whole statocyst is filled with gastrodermis cells that contain the statolith. The same statolith chemical composition (basanite) was found in other Scyphozoan medusa^47^,^48^,^64^. Calcium sulphate hemihydrate is, to date, known only in rophaliophoran medusa. It makes it a very specific feature with an important taxonomic value.

Cnidocyst nomenclature is based on the structure of the tubule and its armature^68^,^69^,^70^. We found only two types of cnidocysts, haplonemes and euryteles covering the marginal lappets and the surface of the *C. tuberculata* and *R. pulmo* rophalium. Haplonemes were egg-shaped, with a regularly coiled tubule inside and had an operculum that split off from the capsule during discharge. In euryteles, which is thought to be related with the function of predation^49^, the capsules opened without the operculum splitting off and comprised round to ovoid capsules containing an irregularly tubule inside^49^. Recent studies identified eurytele and birhopaloid (type II) cnidocysts, having broad and prominent shafts in *C. tuberculata*^68^,^69^,^70^. Specific studies have shown that *Rhizostoma pulmo* has four types of cnidocysts, which according to the classification of Mariscal^71^ were indicated to be heterotrichous microbasic euryteles, holotrichous isorhizas, atrichous a-isorhizas and atrichous-isorhizas^72^. In *Rhizostoma octopus* planula only one cnidocyst type, atrichous haplonema, and heterotrichous microbasic euryteles in polyps, ephyrae, and medusa were observed^49^. The presence of the only two types of cnidocyst in our specimens is probably due to the concentration of our observation to the rhopaliar area because of our interest in the effects of sound in the sensory epithelium of the statocyst.

Our study shows the first ultrastructural images, which characterize some of the changes in the Cnidarians statocyst sensory epithelia after sound exposure. We also show that increased damage takes place with time after sound exposure and it is greatest in the same region where significant hair cell extrusion and loss occur. Intact hair cells maintained the kinocilia structure, whereas damaged hair cells were extruded or missing or presented bent, flaccid or missed kinocilia and stereocilia. The mechanism by which sound induces such damage has yet to be determined. In cephalopods, it was shown that the vibration of the whole animal body exposed to sound, encompassed the vibration of the statolith, which is located in the cephalic cartilage^73^. Here, in the case of medusa, statocysts are directly exposed to the surrounding environment and it could be anticipated that propagating vibrations directly triggers movements of the statoliths induced by the external sensory epithelia, independently of the main body vibration.

In terrestrial vertebrates, exposure to very high sound pressure levels may result in the destruction of sensory hair cells of the inner ear leading to a permanent hearing loss^74^,^75^. The same pathology could be caused by exposure to lower levels for longer periods that also lead to the death of sensory cells^76^. Some research reported damage on sensory cells in ears of some fish species after exposure to pure tones or seismic air gun^77^,^78^,^79^,^80^. Other studies suggest that fish sensory epithelia could regenerate after damaged caused by sound exposure^81^,^82^,^83^,^84^ and evidenced a tonotopic organization in teleost saccule, which is thought to be the primary auditory receptor in this group of fishes^84^. Morphological effects on sensory epithelia could lead to temporary deafness that could result on the fish incapacity to respond to presence of predators and to locate preys and mates. Due the differences in fish species hearing systems, the limited data of precise stimulus (pressure and/or particle velocity) and frequency components of the signals, it is necessary to be extremely cautious when extrapolating results.

Further work is needed to determine whether tonotopic mapping of frequencies takes place in Cnidarians statocyst sensory epithelia, as it was determined in zebrafish^83^ and goldfish^84^ saccule. Several factors could be involved in the mechanism that contribute to tonotopic mapping in the fish saccule: the differential vibration of the otolith in function of the differential frequency^85^, the characteristics themselves of the sensory cells^84^,^86^,^87^, its mode of attachment to the otolithic membrane^88^ and the central processing of the sensory stimulus^88^,^89^,^90^ between others. Similar features were suggested to be vital for sound processing in cephalopods^58^,^60^,^61^, but no tonotopic mapping studies on any invertebrate species has been yet conducted.

In invertebrates, the data of the morphological and physiological effects of noise exposure in sound detection systems are scarce and limited. Our previous works on cephalopods presented the first morphological and ultrastructural evidence of a massive acoustic trauma induced on individuals belonging to four cephalopod species (*S. officinalis, O. vulgaris, L. vulgaris* and *I. coindetii*) by low frequency sound Controlled Exposure Experiments (CEE), in laboratory conditions^35^,^36^,^37^. The consequences of such CEE were permanent and substantial alterations of the sensory hair cells of the statocysts. In laboratory experiments^35^,^36^,^37^, cephalopod statocyst sensory epithelia presented similar lesions to those described here in medusae.

These results support the idea that vibration or sound pressure waves detection is mediated by directionally sensitive ciliary hairs^55^,^91^ located in the cnidarians statocyst. Behaviour observations^92^ suggested that *Aurelia* sp. have a distance sensing ability. Sensitivity to sound pressure waves appears to be the only known sensory system that might participate in distance sensing *Pelagia noctiluca* also appears to have a distance sensing ability that helps them avoiding stranding. Turning and swimming down were also found to be elicited when ebb tides swept *A. labiata* over the gravel bar at the entrance to Roscoe Bay^44^. The effective stimulus may have been tumbling in turbulent water, vibration, or sound pressure waves. Swimming down has to be modulated by stimuli indicating the depth of the water column. Feedback from the statocysts would be needed to guide downward swimming in the turbulent water^24^.

As for the location of the observed damage, it was located on the distal part of the rhopalia, mainly coincident with statocyst position (5–25% of the total rhopaliar length). The lack of any lesion in statocyst control animals, together with the similarity of the injuries found in previous experiments with cephalopods after exposure to the same acoustic stimulus, allow us to conclude on a common cause-to-effect relationship between sound and trauma in all exposed individuals.

In addition with the statocyst sensory system, mechanoreceptors associated with cnidocysts may also contribute to vibration and sound pressure wave sensitivity. Cnidocysts of cnidarians contain mechanoreceptors that along with chemoreceptors modulate the sensitivity of nematocysts. There are neural connections between cnidocysts^93^. Cnidocysts are distributed over most of the surface of *Aurelia sp.* mediating its somatosensory sensitivity^92^. Accordingly, it seems reasonable to suggest that the mechanoreceptors associated with the cnidocysts of *Aurelia sp*. send axons into the central nervous system and sub-serve a somatosensory function^66^. Further studies also needed to determine the effects of sound exposure on cnidocysts and its role on vibration and sound pressure wave detection.

The structure of pelagic ecosystems can change rapidly due to climate change, eutrophication, overfishing, translocations and habitat modification. In these changing habitats, the increasing introduction of noise certainly represents an additional pressure to the ecosystems and it may dramatically affect ocean soundscapes. Regulation is not including marine invertebrates in the list of species that are sensitive to human-generated noise and that need to be considered for mitigation. The presence of acoustic trauma in marine species that are not hearing specialists, like medusa, shows the magnitude of the problem of noise pollution and the complexity of the task to determine threshold values that would help building up management actions to understand and prevent permanent damage of the ecosystems.

## Methods

**Figure 11:**
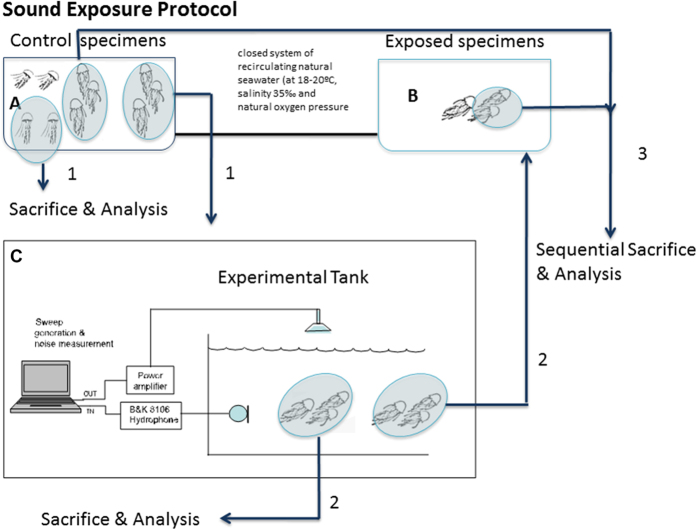
Scheme of the general protocol of the exposure to sound and posterior analyses (Modified from 36).

Twelve individuals from *C. tuberculata* (bell diameter 10–22 cm) and six *R. pulmo* (bell diameter 13–20 cm) were obtained from the Catalan coast (NW Mediterranean Sea) and kept in a closed system of recirculating natural seawater (at 18–20 °C, salinity 35‰ and natural oxygen pressure) consisting of 2 mechanically filtered fiberglass reinforced plastic tanks with a capacity of 2000 L, that were connected to each other (LAB-UPC, Vilanova i la Geltrú) (Fig. 11). This included a physicochemical self-filtration system with activated carbon and sand, driven by a circulation pump. Individuals were maintained in the tank system until the exposure. Several specimens (see below) were used as controls and were kept in the same conditions as the experimental animals until being exposed to noise.

### Sound Exposure Protocol

Sequential Controlled Exposure Experiments (CEE) were conducted on individuals of *C. tuberculata* (n = 8) and *R. pulmo* (n = 4). An additional set of individuals of *C. tuberculata* (n = 4) and *R. pulmo* (n = 2) was used as a control. Individuals were maintained in the tank system (tank A) until the time of exposure. The exposure consisted of a 50–400 Hz sinusoidal wave sweeps with 100% duty cycle and a 1-second sweep period for two hours. The sweep was produced and amplified through an in-air loudspeaker while the level received was measured by a calibrated B&K 8106 hydrophone (RL = 157 ± 5 dB re 1 μPa with peak levels up to SPL = 175 dB re 1μPa). Some of the animals were used as controls and were kept in the same conditions as the exposed animals until the latter were exposed to noise, in an independent tank (C). The sacrificing process was identical for controls and exposed animals. After the exposure, the individuals were placed in tank B (see Fig. 11^36^ and sequence of sacrifices below). The independent experimental tank (C) was located in a separate location, acoustically isolated from tanks A and B. Following exposure, the samples (Fig. 11) were obtained from the individuals (exposed and controls) at 48 h and 96 h after sound exposure.

### Removal of statocysts

In all experiments, the whole rhopalia, containing the statocyst, were detached and chemically fixed for observation and analysis. Between four and eight rhopalia were obtained for each individual depending on its diameter. Each rhopalium was cut open and flattened out to expose the sensory epithelia surrounding the statocyst structures and special care was taken to prevent mechanical damage to the tissues.

### Light microscopy (LM)

Previous to the individual statocyst dissection from the rhopalia some light microscopy images of the whole structure were taken to clarify the morphology and location of the statocyst. The result section will thus concentrate on a qualitative ultrastructural description of the rhopalia sensory structures.

### Scanning electron microscopy

Forty eight statocysts from 12 *C. tuberculata* and 24 statocysts from 6 *R. pulmo* were used for this study. Fixation was performed in 2, 5% glutaraldehyde for 24–48 h at 4 °C. Statocysts were dehydrated in graded ethanol solutions and critical-point dried with CO_2_ in a Bal-Tec CPD 030 unit (Leica Mycrosystems, Austria). The dried rhopalia were cut, open and flattened out to expose the statocyst structures and then mounted on specimen stubs with double-sided tape. The mounted tissues were gold-palladium coated with a Polaron SC500 sputter coated unit (Quorum Technologies, Ltd.) and viewed with a variable pressure Hitachi S3500N scanning electron microscope (Hitachi High-Technologies Co., Ltd, Japan) at an accelerating voltage of 5 kV in the Institute of Marine Sciences of the Spanish Research Council (CSIC) facilities. The chemical composition of the statolith crystals were additionally analysed with a Quantax Energy Dispersive Spectrometer (EDS) (Bruker AXS). The software used to analyse the spectra was Esprit 1.8 (Bruker AXS).

### Quantification and Data analysis

We considered for the analysis the region comprising the sensory areas of the statocyst. The length of the area comprising hair cells was determined for each sample, and 900 μm^2^ (30 × 30 μm) sampling squares were placed along the centre length of the area at 5, 25, 50, and 75% of the length on axe of the statocyst (Figs 2 and 3). The hair cell bundle densities at the most proximal tip of the area (from 75% -in *C. tuberculata*- and 50% -in *R. pulmo*- of length measured) were too high to accurately quantify; therefore, we chose not to attempt quantification of hair bundle density and damage in this area. The area quantified is coincident with the position of the statocyst into the rhopalium.

Hair cell damage was analysed by classifying the hair cells as intact (hair cell undamaged), damaged (kinocilium or surrounding sterocilia partially or entirely missing or fused), extruded (hair cell partially extruded of the epithelium) and missing (hole in the epithelium caused by the total extrusion of the hair cell).

Damage was quantified as the percentage of extruded and missing hair cells. The damaged category included a wide range of different types of lesions with different severities; this makes direct comparison between animals more difficult. The extruded and missing categories are well-defined and are easier compared and the presence of extruded cells shows the limit of severe damage after sound exposure.

Damage due to sound exposure was tested using permutation tests. Data was summed over all regions. Damage was measured as the percentage of extruded and missing hair cells. Permutation tests were repeated multiple times with N = 1000. The influence of recovery time after sound exposure in groups of animals sacrificed 48 h vs 96 h after sound exposure was tested using permutation tests repeated multiple times with N = 1000. The difference in damage between regions was measured as the percentage of extruded and missing hair cells per region 5% vs 25% region at 48 h and 96 h after sound exposure using permutation tests repeated multiple times with N = 1000.

## Additional Information

**How to cite this article**: Solé, M. *et al*. Evidence of Cnidarians sensitivity to sound after exposure to low frequency noise underwater sources. *Sci. Rep.* **6**, 37979; doi: 10.1038/srep37979 (2016).

**Publisher's note:** Springer Nature remains neutral with regard to jurisdictional claims in published maps and institutional affiliations.
