# Corrigendum: Evidence of Cnidarians sensitivity to sound after exposure to low frequency underwater sources

**DOI:** 10.1038/srep43193

**Published:** 2017-02-23

**Authors:** Marta Solé, Marc Lenoir, José Manuel Fortuño, Mercè Durfort, Mike van der Schaar, Michel André

Scientific Reports 6: Article number: 3797910.1038/srep37979; published online: 12 21 2016; updated: 02 23 2017

The original version of this Article contained errors.

The Title,

“Evidence of Cnidarians sensitivity to sound after exposure to low frequency noise underwater sources”

now reads:

“Evidence of Cnidarians sensitivity to sound after exposure to low frequency underwater sources”

The author José Manuel Fortuño, was incorrectly given as José Manuel Fontuño.

In addition, the legend of Figure 5:

“**Figure 5: (A,B,D,E) SEM. (C) EDX analysis. (A) (C) tuberculata statolith. (B) R. pulmo statolith**.

The broken crystal allows to see the inner structure of the statolith. (**C**) **Chemical analysis of statolith** shows the mainly presence of calcium, sulphur and oxygen. Au and Pd peaks appears because sample was previously coated with Au-Pd. **(D)*****R. pulmo*** **discharged eurytele cnidocyst**. The operculum is bent up after discharge of the tubule. The shaft bears long. **Insert in (D)** ***R. pulmo*** **discharged haploneme cnidocyst**. The operculum split off from the capsule during the discharge of the tubule. **(E)** ***C. tuberculata*** **discharged eurytele cnidocyst** (c: capsule, o:- operculum, sh: shaft, la: lamellae, t: tubule). Scale bars: A, D, E = 5 μm. B = 2 μm.”

now reads:

“**Figure 5. A, B, D, E: SEM. C: EDX analysis.**

**(A)** C. tuberculata statolith. **(B)** R. pulmo statolith. The broken crystal allows to see the inner structure of the statolith. (**C**) Chemical analysis of statolith shows the mainly presence of calcium, sulphur and oxygen. Au and Pd peaks appears because sample was previously coated with Au-Pd. (**D**) *R. pulmo* discharged eurytele cnidocyst. The operculum is bent up after discharge of the tubule. The shaft bears long. **Insert in (D)** *R. pulmo* discharged haploneme cnidocyst. The operculum split off from the capsule during the discharge of the tubule. (**E)** *C. tuberculata* discharged eurytele cnidocyst (c: capsule, o- operculum, sh: shaft, la: lamellae, t: tubule). **Scale bars**: **A, D, E** = 5 μm. **B** = 2 μm.”

In addition, the Legend of Figure 9:

“**Figure 9: Mean intact hair cell, damaged hair cell, extruded hair cell and missing hair cell at 5, 25 and 50% of the total length of sensory epithelium on rhopalia of** ***C. tuberculata***.

(A) and at 5 and 25% of the total length of sensory epithelium on rhopalia of *R. pulmo* (**C**) (48 h and 96 h after sound exposure versus control animals). Note the increase of damaged, extruded and missing cells versus controls with increase of time. **Mean (±SE) intact hair cell, damaged hair cell, extruded/missing hair cells after sound exposure versus control on epithelium rhopalia of** ***C. tuberculata*** **(B) (n = 16) and** ***R. pulmo*** **(D) (n = 8).** (**B,D**: Each bar is the average over the 3 (**B**) or 2 (**D**) zones with the line indicating the standard error. The percentage was computed by dividing with the total count for each individual sample.)”

now reads:

“**Figure 9: Mean intact hair cell, damaged hair cell, extruded hair cell and missing hair cell at 5, 25 and 50% of the total length of sensory epithelium on rhopalia of** ***C. tuberculata*** **(A) and at 5 and 25% of the total length of sensory epithelium on rhopalia of** ***R. pulmo*** **(C) (48 h and 96 h after sound exposure versus control animals).**

Note the increase of damaged, extruded and missing cells versus controls with increase of time. **Mean (±SE) intact hair cell, damaged hair cell, extruded/missing hair cells after sound exposure versus control on epithelium rhopalia of** ***C. tuberculata*** **(B) (n = 16) and** ***R. pulmo*** **(D) (n = 8).** (**B,D**: Each bar is the average over the 3 (**B**) or 2 (**D**) zones with the line indicating the standard error. The percentage was computed by dividing with the total count for each individual sample)”.

These errors have now been corrected in the PDF and HTML versions of the Article.

